# The wish to die among palliative home care clients in Ontario, Canada: A cross-sectional study

**DOI:** 10.1186/s12904-016-0093-8

**Published:** 2016-02-29

**Authors:** Shannon Freeman, Trevor Frise Smith, Eva Neufeld, Kathy Fisher, Satoru Ebihara

**Affiliations:** School of Nursing, University of Northern British Columbia, Prince George, British Columbia Canada; Department of Sociology, Nipissing University, North Bay, Ontario Canada; Centre for Rural and Northern Health Research, Laurentian University, Sudbury, Ontario Canada; School of Nursing, McMaster University, Hamilton, Ontario Canada; Department of Rehabilitation Medicine, Toho University Graduate School of Medicine, Tokyo, Japan

**Keywords:** End-of-life, Home care, Hospice palliative care, interRAI, Older adults

## Abstract

**Background:**

In the pursuit to provide the highest quality of person centered palliative care, client preferences, needs, and wishes surrounding end of life should be used to inform the plan of care. During a clinical assessment for care services, clients may voluntarily express a ‘wish to die’ either directly to the clinician or it may be indirectly reported second-hand to the clinician through an informal caregiver or family member. This is the first study using data gathered from the interRAI Palliative Care Assessment instrument (interRAI PC) to examine socio-demographic, clinical, and psycho-social factors of palliative home care clients with the voluntary expression of a ‘wish to die now’. Factors associated with the risk for depression within this group were also identified. Awareness and understanding of clients who express the ‘wish to die’ is needed to better tailor a person-centered approach to end-of-life care.

**Methods:**

This cross-sectional study included assessment records gathered from 4,840 palliative home care clients collected as part of pilot implementation of the interRAI PC assessment instrument in Ontario, Canada from 2006 through 2011.

**Results:**

During the clinical assessment, 308 palliative home care clients (6.7 %) had voluntarily expressed a ‘wish to die now’. Independent factors emerging from multivariate logistic regression analyses predicting the expression of a ‘wish to die’ included not being married/widowed, a shorter estimated prognosis, depressive symptoms, functional impairment, too much sleep (excessive amount), feeling completion regarding financial/legal matters, and struggling with the meaning of life. Among persons who expressed a ‘wish to die now’, those who exhibited depressive symptoms (23.8 %, *n* = 64) were also more likely to exhibit cognitive impairment, have decline in cognition in the last 90 days, exhibit weight loss, have informal caregivers exhibiting distress, ‘not have a consistent positive outlook on life’ and report ‘struggling with the meaning of life’.

**Conclusion:**

When clients voluntary express a wish to die clinicians should take notice and initiate follow-up to better understand the context of this meaning for the individual. Clients who expressed a ‘wish to die’ did not all experience pain, depression, and psychological distress suggesting an individualized approach to care management be taken.

## Background

The aim of palliative care is to reduce pain and suffering through the provision of the highest quality of care available for persons faced with a life limiting illness and members of their circle of care (caregivers, family, and friends). Palliative care’s ‘whole person’ focus includes physical, social, psychological, and spiritual needs. It aims to achieve the highest quality of life (QOL) and death for each person by understanding the meaning and values of the person’s thoughts, feelings, and goals [1].

Expression of the desire to die is not uncommon among persons faced with a life limiting illness [2]. Research in this area has been hindered by methodological challenges [3] and by the paucity of evidence-based clinical practice guidelines to address expressions of wanting to die among persons nearing end of life [4]. Moreover, numerous phrases, including ‘desire for death’, ‘wanting to die’ ‘wish for a hastened death’, ‘wish to die’, ‘euthanasia’, and ‘assisted suicide’, further complicate understanding when examining the epidemiology of the expression [5]. Inconsistencies, overlap, and lack of clarity in meaning of expressions to ‘wish to die’ make research in this area difficult to conduct and moreover pose substantial challenges for researchers to compare research findings among end of life populations [6]. Expressions of a wish to die sometimes but not always equate to a genuine wish to hasten death. Accordingly, expressions for a wish to die should not be taken ‘at face value’ but instead necessitate further discussions to expand understanding of the issues affecting the person which underlie this expression (e.g. the individual’s lived experiences, existential impact of age-related losses, unique temporality, and presence of depressive symptoms [7, 8]).

The prevalence of depression in terminally ill persons is estimated to be between 5 to 30 percent [9]; however, depression at the end of life is not a universal experience among palliative care patients [10]. Depression presents atypically among older adults in general and may mimic common medical conditions that lead to under-diagnosis and under-treatment [11, 12]. These same challenges present among older persons faced with a life limiting illness [13, 14]. Identifying and treating depressive symptoms, which may be an underlying factor to a patient’s expression to want to die now, may improve QOL as the person nears end of life [15].

During the palliative care planning process, clients are encouraged to share their feelings, hopes, and preferences to inform their goals of care and to prioritize needs to be addressed with care team members such as case managers, social workers, nurses, and care staff. During these discussions clients may be encouraged to express their thoughts and wishes surrounding the death and dying process. In contrast to previous research by Ohnsorge [16] which sought a more in-depth understanding of the ‘why’ behind expressions a wish to die now, this study focused on identifying ‘who’ expresses a wish to die now and aimed to identify ‘what’ socio-demographic, clinical, and psycho-social factors were associated with of persons who expressed this wish.

Using quantitative methods, the purpose of this study was to better understand profiles of a sample of palliative clients receiving home care services who voluntarily expressed the wish to want to die now. This study further explored the members of the sample who expressed the ‘wish to die now’ to identify the factors associated with the risk for depression within this group. This is the first study to examine palliative home care clients who expressed a wish to want to die now using the interRAI Palliative Care Assessment instrument (interRAI PC) [17] (http://www.interrai.org/palliative-care.html) .

## Methods

### Data source

Cross sectional data included assessment records from 4,840 palliative home care clients assessed with the interRAI PC assessment instrument. Data were collected during a pilot of the interRAI PC across six regional jurisdictions in Ontario, Canada from 2006 through 2011.

### Measures

The interRAI PC is a comprehensive standardized assessment instrument that gathers information on physical, cognitive, and social domains, as well as demographic, health service utilization, and care preferences to provide a comprehensive description of the client to inform the care planning process [17, 18]. Self-reported information on mood, life-completion, spirituality, informal supports, and advanced directives is also captured. Trained assessors complete the interRAI PC as part of regular clinical practice using information from direct observations, available medical records, and discussions with the client, members of their health care team, and informal support networks. The interRAI PC shows strong inter-rater and test-retest reliability [18, 19].

The outcome measure, the voluntary expression of ‘wish to die now’, heretofore referred to as an expression of a WTD, was dichotomous and reflected the assessor’s judgement on whether or not the client voluntarily expressed the wish to ‘want to die now’, to family members, friends, or staff members [17]. Clinicians are instructed not to probe or directly solicit this information. Therefore this information had to be voluntarily shared with the clinician by the client or by a member of the person’s circle of care (e.g. family member, informal caregiver, or friend) during the clinical assessment conducted for care planning and service allocation purposes.

The Depression Rating Scale (DRS), an indicator of client risk of depression, is calculated using seven mood and behaviour items contained within the interRAI PC [20]. The DRS ranges from 0-14 where a score of 0-2 indicates no to minimal risk of depression while a score of 3-5 indicates moderate risk of depression and a score of 6 or greater indicating high risk of depression. In addition to being validated against the Hamilton Depression Rating Scale and the Cornell Scale for Depression and Dementia [20], the validity and reliability of the DRS have been demonstrated in this palliative home care population [21]. Acceptable levels of internal consistency have been reported in samples across complex continuing care hospitals (α = 0.74), nursing home residents (α = 0.85), and among inpatient mental health patients (α = 0.74) [22–24].

Clinical summary scales, embedded within the interRAI PC, also include: Changes in Health, End-stage disease, and Signs and Symptoms (CHESS) scale, a measure of health instability ranging from 0 (no instability in health) to 6 (highly unstable health) [25]; Cognitive Performance Scale (CPS), measuring level of cognitive ability ranging from 0 (cognitively intact) to 6 (severely cognitively impaired) [26]; Activities of Daily Living Hierarchy Scale (ADL-H) measuring physical functioning ranging from 0 (independent) to 6 (totally dependent) [27]; and the Pain Scale, ranging from 0 (no pain) to 4 (excruciating pain) [28]. Further details on these and other embedded measures within the interRAI PC are available at www.interRAI.org.

### Analysis

Analyses were performed using SAS Version 9.2. This analysis was conducted in two phases. Phase one used bivariate analyses (chi-square and t-tests depending on variable type) to determine significance between the outcome (WTD) and each co-variate to profile sociodemographic, clinical, and psychosocial characteristics of persons who expressed a WTD compared to the palliative care population who did not express a WTD. An alpha level of *p* < 0.05 was set for all bivariate analyses. Bivariate analyses informed multivariate logistic regression analyses that used the dichotomous outcome (WTD – Yes/No) as the dependent variable and the socio-demographic, clinical and psychosocial characteristics as independent variables. Co-variates, significant at the bivariate level using a relaxed level of significance (*p* = 0.20), were included in the modeling. At later stages, this was then increased to *p* = 0.05 for inclusion in the final model. Polychoric correlation coefficients were examined to identify multicolinearity concerns, with a cutoff of >0.5 used to identify variables to be excluded from regression modeling process. Goodness-of-fit of the final logistic regression model was determined using Hosmer and Lemeshow’s Goodness-of-Fit test with *p* >0.05 [29]; while the c-statistic was used to determine model strength [30]. A phase two sub-analysis focused only on clients who expressed a WTD (*n* = 268) and compared differences among this group between those who exhibited depressive symptoms and those who did not exhibit depressive symptoms.

Review by the University of Northern British Columbia’s Institutional Research Ethics Board determined this study did not require ethics approval as it used de-identified secondary data. Additional informed consent was not required for this study as the interRAI palliative care assessment instrument was used as part of routine clinical practice. All personal identifiers are removed from the data prior to their submission to interRAI Canada.

## Results

Socio-demographic, clinical, and psychosocial characteristics describing palliative home care clients (with and without the WTD) who accessed palliative home care services in Ontario, Canada, are shown in Table 1. Clients were predominantly over the age of 65 (66.4 %) with balanced representation by sex. The prevalence of expressing a WTD was 7 per 100 palliative home care clients. The proportion of clients who expressed a WTD increased with age. Clients over the age of 85 years were three times more likely to express a WTD (OR 3.09, 95 % CI 2.19-4.36) when compared to clients under the age of 65. While no significant differences were observed by sex, those who were married were less likely to express a WTD (OR 0.65, 95 % CI 0.51-0.82). The estimated prognosis of 90 % of clients was six weeks or greater. Among clients whose prognosis for death was imminent, the odds of WTD was fifteen times higher compared to clients with an estimated prognosis of six months or greater (15.09, 95 % CI 8.73-26.1).

**Table 1:** Key Characteristics Stratified by Clients Expressions of a Wish to Die among Palliative Home Care Clients in Ontario, Canada (*N* = 4,840)

		Total Population (*n* = 4,840)	Expressed Wish to die (6.7 %, *n* = 308)	Did Not Express Wish to Die (93.3 %, *n* = 4,532)	Unadjusted Odds Ratio (95 % CI) ^ŧ^
Sociodemographic characteristics					
Age Group ^****^	18-64^a^	33.6 (1,628)	22.1 (68)	34.4 (1,560)	1.00
	65-74	25.3 (1,222)	21.1 (65)	25.5 (1,157)	1.29 (0.91-1.83)
	75-84	28.6 (1,383)	33.4 (103)	28.2 (1,280)	1.85 (1.35-2.53)
	85 +	12.5 (607)	23.4 (72)	11.8 (535)	3.09 (2.19-4.36)
Gender	Male^a^	49.3 (2,367)	44.9 (136)	49.5 (2,231)	1.00
	Female	50.8 (2,439)	55.1 (167)	50.5 (2,272)	1.21 (0.95-1.52)
Marital Status^**^	Not Married^a^	37.2 (1,748)	47.1 (140)	36.5 (1,608)	1.00
	Married/Have Partner	62.8 (2,952)	52.9 (157)	63.5 (2,795)	0.65 (0.51-0.82)
Clinical characteristics					
Prognosis^****^	Death Imminent	1.9 (75)	10.4 (26)	1.3 (49)	15.09 (8.73-26.1)
	Less than 6 weeks	9.3 (364)	23.1 (58)	8.4 (306)	5.39 (3.65-7.97)
	6 weeks to 6 months	48.1 (1,879)	45.0 (113)	48.3 (1,766)	1.82 (1.31-2.54)
	Greater than 6 months^a^	40.7 (1,590)	21.5 (54)	42 (1,536)	1.00
Reported Diagnosis^****^	Cancer Only^a^	61.6 (2,855)	61.0 (178)	61.6 (2,677)	1.00
	Non-Cancer Only	10.4 (480)	18.2 (53)	9.8 (427)	1.87 (1.35-2.58)
	Cancer & Non-Cancer	28.1 (1,301)	20.9 (61)	28.6 (1,240)	0.74 (0.55-0.99)
Functional Impairment^****^	No-minimal (ADL-H = 0)^a^	48.6 (2,223)	25.1 (71)	50.1 (2,152)	1.00
	Mild-moderate (ADL-H = 1-2)	23.2 (1,064)	18.7 (53)	23.5 (1,011)	1.59 (1.11-2.29)
	Severe (ADL-H ≥ 3)	28.2 (1,291)	56.2 (159)	26.4 (1,132)	4.26 (3.19-5.68)
Decline in Functional Ability^****^	No^a^	20.9 (906)	7.8 (23)	21.9 (883)	1.00
	Yes	79.1 (3,420)	92.2 (272)	78.1 (3,148)	3.32 (2.15-5.11)
Cognitive Impairment^****^	No-minimal (CPS = 0) ^a^	66.8 (3,087)	52.3 (146)	67.7 (2,941)	1.00^a^
	Mild-moderate (CPS = 1-2)	26.7 (1,234)	30.1 (84)	26.5 (1,150)	1.47 (1.12-1.94)
	Severe (CPS ≥ 3)	6.5 (301)	17.6 (49)	5.8 (252)	3.92 (2.765-5.55)
Decline in Cognition^****^	No^a^	72.1 (3,127)	56.0 (158)	73.2 (2,969)	1.00
	Yes	27.9 (1,210)	44.0 (124)	26.8 (1,086)	2.15 (1.68-2.74)
Health Stability^****^	Stable (CHESS = 0-1)	16.0 (709)	6.3 (18)	16.6 (691)	-
	Unstable (CHESS = 2-3)	47.1 (2,091)	36.3 (103)	47.9 (1,988)	-
	Highly Unstable (CHESS = 4-5)	36.9 (1,637)	57.4 (163)	35.5 (1,474)	-
Depressive Symptoms	No-Minimal Risk (DRS = 0-2) ^a^	91.9 (4,200)	76.2 (205)	92.9 (3,995)	1.00
	Moderate Risk (DRS =3-5)	6.4 (291)	14.9 (40)	5.8 (251)	3.11 (2.16-4.46)
	High Risk (DRS ≥ 6)	1.8 (80)	8.9 (24)	1.3 (56)	8.35 (5.07-13.75)
Pain^*^	None (PS = 0) ^a^	26.3 (1,185)	26.0 (73)	26.3 (1112)	1.00
	Less than severe (PS = 1-2)	55.5 (2,502)	49.8 (140)	55.9 (2,362)	0.90 (0.67-1.21)
	Severe-excruciating (PS = 3-4)	18.2 (822)	24.2 (68)	17.8 (754)	1.37 (0.98-1.94)
Breakthrough Pain^*^	No^a^	66.1 (3,083)	59.4 (174)	66.5 (2,909)	1.00
	Yes	33.9 (1,582)	40.6 (119)	33.5 (1,463)	1.36 (1.07-1.73)
New Pain^****^	No^a^	88.9 (4,110)	81.1 (227)	89.4 (3,883)	1.00
	Yes	11.1 (515)	18.9 (53)	10.6 (462)	1.96 (1.43-2.69)
Fatigue^****^	No^a^	19.0 (901)	8.3 (25)	19.7 (876)	1.00
	Yes	81.1 (3,854)	91.7 (275)	80.3 (3,579)	2.69 (1.78-4.08)
Weight loss^**^	No^a^	52.8 (2,421)	43.9 (127)	53.4 (2,294)	1.00
	Yes	47.2 (2,162)	56.1 (162)	46.6 (2,000)	1.46 (1.15-1.86)
Bladder Incontinence^****^	No^a^	82.5 (3,948)	70.3 (213)	83.3 (3,735)	1.00
	Yes	17.5 (840)	29.7 (90)	16.7 (750)	2.10 (1.63-2.73)
Too Much Sleep^****^	No^a^	77.8 (3,607)	51.2 (148)	79.5 (3,459)	1.00
	Yes	22.2 (1,031)	48.8 (141)	20.5 (890)	3.70 (2.91-4.72)
Psychosocial characteristics					
Sense of completion on financial, legal, & formal responsibilities^****^	No^a^	20.2 (929)	11.1 (32)	20.8 (897)	1.00
	Yes	79.8 (3,676)	88.9 (257)	79.2 (3,419)	2.11 (1.45-3.07)
Has strengths that can be fostered^****^	No^a^	6.4 (298)	13.5 (39)	5.9 (259)	1.00
	Yes	93.6 (4,385)	86.5 (249)	94.1 (4,136)	0.40 (0.28-0.57)
Consistent positive outlook ^****^	No^a^	20.2 (939)	44.6 (124)	18.6 (815)	1.00
	Yes	79.8 (3,712)	55.4 (154)	81.4 (3,558)	0.28 (0.22-0.37)
Struggling with meaning of life^****^	No^a^	89.5 (3,503)	77.5 (162)	90.2 (3,341)	1.00
	Yes	10.5 (409)	22.5 (47)	9.8 (362)	2.68 (1.90-3.77)
Informal Caregiver Exhibits Distress^****^	No^a^	75.9 (3,460)	65.1 (190)	76.6 (3,270)	1.00
	Yes	24.1 (1,100)	34.9 (102)	23.4 (998)	1.76 (1.37-2.26)

Note: CI denotes confidence interval; ADL-H denotes Activities of Daily Living Hierarchy Scale; CPS denotes Cognitive Performance Scale; DRS denotes Depression Rating Scale; PS denotes Pain Scale; * *p* < 0.05 ** *p* < 0.01 ***p < 0.001 **** *p* < 0.0001

^a^Reference Groups ^ŧ^Modeled odds that client expressed a wish to die (WTD)

Approximately two thirds of clients reported only a cancer diagnosis (61.6 %). Clients with only a non-cancer diagnosis were significantly more likely to have expressed a WTD (OR 1.87 95 % CI 1.35-2.58) compared to clients with only a cancer diagnosis. Clients with multiple cancer and non-cancer diagnoses were less likely to have expressed a WTD (OR 0.74, 95 % CI 0.55-0.99) when compared to clients with only a cancer diagnosis.

A breakdown of cancer and selected non-cancer diagnoses are shown in Table 2. Cancer diagnoses were grouped according to International Classification of Disease (ICD-10) [31]. The most commonly reported cancers were cancer of the respiratory and intrathoracic organs (26.1 %); cancer of the digestive organs (25.4 %); and cancer of the breast (9.2 %). Of non-cancer diagnoses, the most commonly reported diagnoses were diabetes (10.0 %); hypertension (8.1 %); COPD (5.6 %); and congestive heart failure (4.1 %6). Alzheimer’s and dementia’s other than Alzheimer’s disease were not commonly reported (2.5 %, *n* = 112).

**Table 2:** Key Characteristics Stratified by Clients Expressions of a Wish to Die among Palliative Home Care Clients in Ontario, Canada (*N* = 4,840)

	Total Population (*n* = 4,840)	Did Not Express Wish to Die (93.3 %, *n* = 4,532)	Expressed Wish to die (6.7 %, *n* = 308)
Non-Cancer diagnosis (selected)			
Diabetes	10.0 (483)	9.9 (449)	11.0 (34)
Hypertension*	8.1 (393)	8.3 (378)	4.9 (15)
COPD	5.6 (271)	5.5 (249)	7.1 (22)
Congestive Heart Failure**	4.1 (196)	3.8 (173)	7.5 (23)
Coronary Artery Disease	3.2 (153)	3.2 (144)	2.9 (9)
Renal Failure	2.7 (131)	2.7 (121)	3.2 (10)
Alzheimer’s and other Dementia’s	2.5 (112)	2.3 (106)	5.2 (16)
Cerebrovascular Accident***	1.8 (87)	1.6 (74)	4.2 (13)
Liver Disease	2.2 (107)	2.2 (101)	2.0 (6)
Gastrointestinal Disease	1.7 (84)	1.8 (80)	1.3 (4)
Cancer (by primary specified site)			
Respiratory and Intrathoracic**	26.1 (1,265)	26.7 (1,208)	18.5 (57)
Digestive Organs	25.4 (1,231)	25.5 (1,157)	24.0 (74)
Breast	9.2 (444)	9.3 (421)	7.5 (23)
Male Genital	6.0 (288)	6.0 (270)	5.8 (18)
Lymphoid, Hematopoietic, and Related Tissue	5.3 (256)	5.3 (239)	5.5 (17)
Urinary Tract	4.6 (221)	4.6 (210)	3.6 (11)
Female Genital	4.5 (218)	4.5 (203)	4.9 (15)
Eye, Brain and Central Nervous System	3.7 (177)	3.7 (168)	2.9 (9)
Ill-defined, Secondary, Unspecified Sites	3.5 (170)	3.5 (159)	3.6 (11)
Skin	2.3 (112)	2.3 (103)	2.9 (9)
Lip, Oral, and Pharynx**	2.0 (95)	1.8 (83)	3.9 (12)
Mesothelial and Soft Tissue	1.9 (90)	1.9 (84)	2.0 (6)
Bone and Articular Cartilage	1.0 (47)	1.0 (45)	0.7 (2)
Thyroid and Endocrine Glands	0.4 (19)	0.4 (18)	0.3 (1)
Independent (Primary) Multiple Sites	0.1 (3)	0.04 (2)	0.3 (1)

Note: * *p* < 0.05 ** *p* < 0.01 ***p < 0.001

Overall, symptoms of depression were not commonly reported among clients with only 6.4 % exhibiting moderate risk of depression (DRS score between 3 and 5) and 1.8 % exhibiting high risk of depression (DRS score of 6 or greater). However, among clients who expressed a WTD, 23.8 % exhibited symptoms of depression compared to only 7.1 % of clients who did not express a WTD. Clients at moderate risk of depression were 3.11 times more likely to have expressed a WTD compared to clients not at risk of depression (DRS score between 0 and 2). Clients at high risk of depression were 8.35 times more likely to have expressed a WTD than clients not at risk of depression (DRS score between 0 and 2).

Clients who exhibited functional and cognitive impairment, increased health instability, or who reported experiencing negative symptoms including fatigue, weight loss, bladder incontinence, and too much sleep (excessive amount of sleep that interferes with person’s normal daily functioning [17]) were all significantly more likely to have expressed a WTD. Three quarters of clients (73.7 %) reported experiencing pain with one in four exhibiting severe to excruciating pain (18.2 %; Pain Scale 3-4); however pain as measured by the Pain Scale was not significantly associated with increased odds to express a WTD. Recent changes in pain (new pain or worsening pain in the last 3 days), decline in cognitive and functional abilities (as compared to 90 days or since last assessment) and experiencing breakthrough pain (in the last 3 days) all emerged as significant risk factors associated with increased likelihood to express a WTD.

All psychosocial characteristics emerged as strong protective and risk factors (*p* < 0.0001 for all psychosocial characteristics shown in Table 1). Clients who had expressed ‘a sense of completion regarding financial, legal and formal matters’, were ‘struggling with a meaning of life’ or who had a caregiver that exhibited signs of distress were all more likely to have expressed a WTD. In contrast, clients who reported having ‘strengths that can be fostered’ or who had ‘consistent positive outlook on life’ were less likely to have expressed a WTD.

In the logistic regression model, characteristics that remained significantly associated with the expression of a WTD included: being married or having a partner, shorter length of estimated prognosis, increased risk of depression, severe functional impairment, too much sleep, having a sense of completion in regards to financial responsibilities, and struggling with the meaning of life (Table 3). Interestingly age (*p* > 0.0001), pain (*p* < 0.05), weight loss (*p* < 0.01), bladder incontinence (*p* < 0.0001), and caregiver distress (*p* < 0.0001), were all significant at the bivariate level, yet did not emerge as strong significant factors in the final multivariate logistic regression model. Similarly, variables indicating recent change in health status including decline in cognition (*p* > 0.0001), decline in physical functioning (*p* > 0.0001) as well as presence of new pain (*p* > 0.0001) or breakthrough pain (*p* > 0.05) in the last 3 days; that were all statistically significant at the bivariate level did not emerge statistically significant predictors in the final multivariate logistic regression model. A c statistic in the final model of 0.78 indicated acceptable predictive strength of this model while a relatively small Hosmer and Lemeshow statistic along with a large p value (6.69; *p* = 0.57) indicated acceptable model fit.

**Table 3:** Logistic Regression Model Predicting Persons Who Express the Desire to Want to Die Now at Time of Baseline Assessment, Ontario Palliative Home Care Clients 2006-2011, Ontario, Canada (*N* = 2,754)

Independent Variable	Parameter estimate (SE)	Adjusted odds ratio (95 % CI)	*p* value
Marital Status (Ref = Married/Have partner)			
• Not Married/Widowed	0.86 (0.18)	2.36 (1.64-3.38)	<0.0001
Prognosis (Ref = 6 months or greater)			
• Death is imminent	1.93 (0.43)	6.92 (2.96-16.18)	<0.0001
• Less than 6 weeks	0.89 (0.29)	2.44 (1.38-4.30)	0.002
• Greater than 6 weeks but less than 6 months	0.22 (0.23)	1.25 (0.80-1.95)	0.32
Depressive Symptoms (Ref = No-Minimal Risk (DRS = 0-2))			
• Moderate risk (DRS = 3-5)	1.08 (0.28)	2.94 (1.70-5.10)	0.0001
• High risk (DRS ≥ 6)	1.25 (0.47)	3.50 (1.40-8.75)	0.007
Functional Ability (Ref = No-minimal (ADL-H = 0))			
• Mild-moderate (ADL-H = 1-2)	0.03 (0.28)	1.03 (0.60-1.77)	0.92
• Severe (ADL-H ≥ 3)	0.95 (0.22)	2.59 (1.67-3.99)	<0.0001
Too Much Sleep (Ref = No)			
• Yes	0.78 (0.20)	2.18 (1.48-3.21)	<0.0001
Has Sense of Completion on Financial, Legal, and Other Formal Responsibilities (Ref = No)			
• Yes	1.23 (0.36)	3.43 (1.69-6.96)	0.0006
Struggling with the Meaning of Life (Ref = No)			
• Yes	0.6 (0.26)	1.83 (1.10-3.04)	0.02

Note: SE denotes standard error; CI denotes confidence interval

C statistic 0.78

Hosmer and Lemeshow x^2^ = 6.69, df = 8, *p* = 0.57

**Fig. 1:**
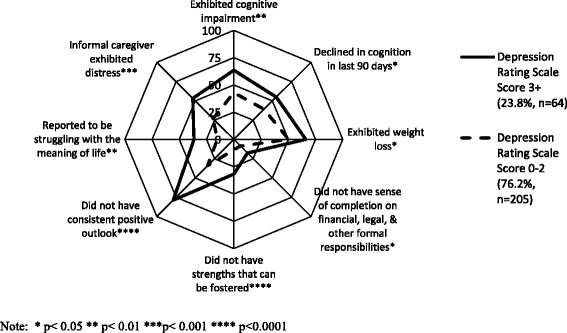
Key Characteristics of Persons who Expressed Wish to Die Now Stratified by Scores on the Depression Rating Scale among a sample of palliative homecare clients in Ontario, Canada (*N* = 269)

Clients who expressed a WTD were examined in closer detail stratified by whether or not they exhibited symptoms of depression (DRS 3 or greater versus DRS 0-2). Among the 308 clients who expressed a WTD, a DRS score was not available for 39 clients; of the 269 clients for whom a DRS score was available, 23.8 % exhibited symptoms of depression (*n* = 64). No significant differences were observed in sociodemographic characteristics nor by the majority of clinical characteristics including prognosis, diagnosis, functional impairment, and pain between those who exhibited/did not exhibit symptoms of depression (data not shown). Among clients who expressed a WTD, 71.9 % of those exhibiting symptoms of depression had highly unstable health (CHESS Score = 4-5) compared to 54.4 % of those not exhibiting symptoms of depression. When examining clinical characteristics of clients who expressed a WTD and comparing clients who exhibited/did not exhibit symptoms of depression, those clients who did exhibit depressive symptoms were more likely to exhibit cognitive impairment (63.5 % vs. 42.9 %), decline in cognition in the last 90 days (55.0 % vs. 39.2 %), and weight loss (66.1 % vs. 50.3 %) (Fig. 1). In addition, persons who expressed a WTD and exhibited symptoms of depression were also more likely to be struggling with the meaning of life (36.6 % vs. 15.2 %), not express a sense of completion regarding financial issues (17.7 % vs. 8.5 %), not perceive they had strengths which could be fostered (31.8 % vs. 9.5 %), not have a consistent positive outlook on life (78.7 % vs. 34.0 %) and have informal caregivers experiencing distress (53.2 % vs. 26.9 %).

## Discussion

Findings highlight a multidimensional picture of clients who express a WTD. Some clients who expressed a WTD showed elevated levels of psychological distress, depression, and pain; while others showed less functional and cognitive impairment and reported ‘strengths which can be fostered’ and a ‘positive outlook on life’. Previous research has suggested it would be inappropriate to reduce WTD statements to symptoms, and instead better to approach expressions of WTD as communicative acts with multiple intentions, reasons, meanings, and possible functions [16, 32]. Similarly, the multidimensional profiles of clinical characteristics of persons who expressed a WTD in our study do not support a uniform approach to care. Instead findings suggest clinicians providing palliative care to the ‘whole’ person may consider to embrace and not fear person-specific conversations surrounding the client’s preferences for death as not all clients expressing a WTD were depressed and showing signs of distress. As shown in similar studies [16] client initiation of a conversation around WTD should not be viewed as a statement but instead should be perceived as the beginning of a narrative discussion surrounding the client’s self-perceived need to express their feelings and communicate the meaning of what an expression of WTD means to them.

The prevalence of persons receiving palliative home care services who expressed a WTD was very small (6.9 %), similar to the prevalence of 7.3 % reported by Güell, Ramos, Zertuche and Pascual [5] who also relied upon clients to voluntarily express this desire in their cross-sectional study of cancer patients receiving palliative care. Other studies that utilized wish to hasten death scales [33, 34], reported increased prevalence of 14 % to 17 % [35, 36]. This difference in prevalence is likely related to the measure of WTD used in the current study which relied on voluntary expression of WTD without any prompting, encouragement, or being directly asked by the assessor during the care planning process. As such, it is likely that this study greatly underestimates the number of palliative home care clients who WTD. This has also been previously suggested by Hudson [4] who suggested that if patients were encouraged to express their wishes then the incidence of expressions of WTD may increase. Hudson [4] found only 2 % of clients with cancer had discussed assisted suicide while when asked, 27 % had thought about assisted suicide. This should provide some consolation for clinicians hesitant to initiate discussion as not all persons who express a WTD will then request assisted suicide.

Expressing a WTD in this study did not always signal a situation of excruciating pain, or deep psychological distress and therefore may not necessarily equate to an expression for hastened death. Similar to the study by Nissim [32], our findings reflected multiple distinctions between client profiles supporting that some expressions of a WTC reflected symptoms of depression and despair, while others represented preparations for death and being at peace. In the current study pain and other physical clinical characteristics including fatigue and incontinence did not emerge at the multivariate level as strong predictors of expressions of a WTD. Instead, our study found a strong association between expressions of despair relating to risk of depression and psychosocial distress. The multivariate analyses in the current study support previous research that found marital status, depression, and drowsiness were strongly associated with an increased likelihood to express a WTD [3]. This research also found age, sex, and pain not to be strongly associated with expression of a WTD [3]. This contrasts the major finding by Emmanual and Fairclough [37] and Güell, Ramos, Zertuche and Pascual [5] of pain being one of the most common reasons for expressing a desire to die. However, Emmmanual and Fairclough [37] also noted symptoms of depression and unmet needs to be equally strong. In our analysis, pain did not emerge as the strongest predictive factor. Instead, depression, second to estimated prognosis, emerged as a very strong predictive factor where persons at high risk of depression were more than eight times more likely to express a WTD at the bivariate level and more than 3 times more likely at the multivariate level. It is recommended that when a client expresses a WTD, clinicians consider screening for depression which may have been previously undiagnosed [38] O’Mahoney et al., found that improvement in depression among a group of patients with cancer was predictive to moderate the severity of desire for a hastened death [38]. Accordingly, psychological symptom management and mental health care may comprise an important component of optimal palliative care [39].

Although a WTD may be a response to suffering, findings in this study suggest this is not a universal experience among persons who express a WTD. Over three quarters of clients who expressed a WTD (76.2 %) did not show symptoms of depression. In addition, persons who expressed a WTD were also more likely to experience a sense of completion regarding financial matters and a large proportion also perceived they had strengths which could be fostered. Over half reported having a consistent outlook on life. These findings suggest that among the population of persons who express a WTD, for some, this may reflect an acceptance and preparation for death rather than a response to uncontrolled pain, distress, and suffering.

An alternative explanation warranting attention and further investigation is the strong relationships between variables which show recent changes in status and increased likelihood to express a WTD. Future research is needed to investigate the temporal relationship between recent changes in physical and cognitive functioning as well as new pain and expression of WTD. Perceived change in life trajectory after diagnosis with a life limiting illness, and potentially as one progresses in the disease process, can become a serious threat to the viability of the person’s hopes, dreams and aspirations for the future [1]. As the illness process progresses and as people age, changes in the person’s abilities may affect their self-perception of health and QOL, as well as challenge their perceived role in society and relationships with others [1]. It is possible that these changes may also affect the person’s expressions of a WTD. Changes may be perceived as negative, for example, financial insecurity and decreased or lost productivity [40, 41]; or as positive, for example, motivation to resolve unfinished business or reconcile past relationships, promote recognition of inner resilience [1] or a combination of both. Care planning involving the person as part of the process is needed to address defined problems on a person-by-person basis that require immediate care interventions without which the problem may worsen [42]. Recognizing the relationship between recent decline and changes in functioning with expressions of WTD are important. Conversations surrounding how the client is adjusting to these changes are critical components to inform person-centered care planning.

The overall proportion of risk of depression reported in this study was low (8.1 %). In previous work, it was evident that institutionalized older adults with depression symptoms often present in atypical ways and that depression symptoms may be under recognized and underreported [43]. Among persons who expressed a WTD, the proportion of risk of depression was 23.8 %; nearly three times higher than the palliative home care population who did not express a WTD. These rates are substantially higher than the 11.2 % prevalence reported by Ferrand [44] among a population of patients who had expressed a request for a hastened death. This suggests that among approximately one in four palliative home care patients, the expression of a WTD may be a feature of the depression symptoms. The low proportion of depression symptoms in the overall study sample may be an underestimate.

In the current study, too much sleep was significantly associated with expressions of a WTD at the bivariate and multivariate levels. Fatigue, difficulty focusing and other physical conditions are often the initial manifestations of depression among older adults [45] and often occur alongside other medical conditions in later life [46]. Depression symptoms should not be regarded as an acceptable phenomenon at the end of life. A wide range of treatments for depression for persons nearing end-of-life are available including pharmaceutical (e.g. anti-depressants, psychostimulants) and non-pharmacologic (e.g. psychotherapy, dignity therapy) treatments [47].

A major challenge to the current dataset is that it relied on persons who willingly initiated this conversation where the client or a person close to them shared the information with the clinician without being asked or probed. In this situation, we believe our findings are representative of a select group of persons nearing end of life who wish to die now and may not reflect the feelings of the larger population of persons nearing end of life. It is possible that persons who volunteer this information without being probed differ from persons who also embody the feelings of wishing to die now but who do not voluntarily verbalize them to others. However, as suggested by Rosenfeld et al. [48], interventions which are pro-active to target persons at potentially elevated risk of WTD are valuable to help reduce future occurrences. In this way, better understanding of clinical profiles of clients who express a WTD may be useful in clinical practice to help target persons who may be at risk and with whom to initiate this conversation.

One of the pillars of palliative care is high quality pain control, which is not solely limited to physical pain but also includes social and psychological pain or existential pain. A limitation of the current analyses regarding pain as captured by the interRAI PC is that the Pain Scale specifically measures physical pain and not existential pain. The current analysis used self-reported mood items as proxy measures to capture psychological and existential distress however it may not be a clear reflection. It is suggested that future revision of the interRAI PC instrument or its associated Clinical Assessment Protocols (CAPs) [49], consider development of more specific measures to capture existential pain as well as an existential pain CAP that uses international best practice guidelines.

Inspiration to write this paper came from discussions with local front line palliative care clinicians who expressed discomfort in initiating conversations about WTD especially during their initial assessment. Clinicians may be worried that initiating conversations surrounding WTD could lead their clients to want to attempt suicide or find means to hasten their death. However, findings from this study dispel this assumption and help inform a clinical picture of which clients are more likely to express a WTD. Using descriptions of needs as generated from the interRAI-PC, clinicians may be able to recognize similar client patterns which they can use as a foundation to initiate end of life conversations with the person that help prioritize client need and identify areas for further assessment and intervention. This study represents one of the largest datasets available focusing on palliative home care clients in Ontario, Canada. Use of interRAI PC assessment data provided a comprehensive picture of palliative home care clients in Ontario, Canada [50]. Future studies using data from the interRAI PC, now that it has been provincially mandated for use across Ontario will enhance opportunity for longitudinal study.

## Conclusions

Findings suggest there are multiple factors associated with person’s expressions of a WTD. If clinicians see a pattern where clients are widowed, have a shorter estimated prognosis, at moderate to high risk of depression, exhibiting severe functional impairment, experiencing too much sleep, have a sense of financial completion, and who are struggling with the meaning of life then they should use their best judgment and consider initiating a conversation about the person’s wishes as they near the end of life. If a client exhibits moderate to severe risk of depression or are sleeping too much then the clinician may also probe into the clients psychosocial characteristics and consider whether treating depression symptoms may affect or change the client’s preference to WTD.

Increased media attention addressing supporting person-centered preferences at the end of life and more specifically, reports discussing right to die, assisted suicide, and euthanasia, further complicate the navigation of this topic. There is a clear need for further research studies (quantitative, qualitative, and mixed methods) to better understand the WTD within the Canadian context. Establishing a foundational understanding of client profiles will allow for more informed attention and recognition by clinicians, which then in turn may lead to more accurate targeting of clients who could benefit from end of life discussions and psycho-social support.

## References

[1] Canadian Hospice Palliative Care Association. A model to guide hospice palliative care: Based on national principles and norms of practice. Revised and condensed edition. Ottawa; 2013. [http://www.chpca.net/media/319547/norms-of-practice-eng-web.pdf.]

[2] Hendry M, Pasterfield D, Lewis R, Carter B, Hodgson D, Wilkinson C (2013). Why do we want the right to die? A systematic review of the international literature on the views of patients, carers and the public on assisted dying. J Palliat Med.

[3] Julião M, Barbosa A, Oliveira F, Nunes B (2013). Prevalence and factors associated with desire for death in patients with advanced disease: results from a Portuguese cross-sectional study. Psychosomatics.

[4] Hudson PL, Kristjanson LJ, Ashby M, Kelly B, Schofield P, Hudson R (2006). Desire for hastened death in patients with advanced disease and the evidence base of clinical guidelines: a systematic review. J Palliat Med.

[5] Güell E, Ramos A, Zertuche T, Pascual A (2015). Verbalized desire for death or euthanasia in advanced cancer patients receiving palliative care. Palliat Support Care.

[6] Monforte-Royo C, Villavicencio-Chávez C, Tomás-Sábado J, Mahtani-Chugani V, Balaguer A (2012). What lies behind the wish to hasten death? A systematic review and meta-ethnography from the perspective of patients. PLoS One.

[7] Mak YYW, Elwyn G (2005). Voices of the terminally ill: uncovering the meaning of desire for euthanasia. J Palliat Med.

[8] van Wijngaarden E, Leget C, Goossensen A (2014). Experiences and motivations underlying wishes to die in older people who are tired of living: a research area in its infancy. Omega (Westport).

[9] Mitchell AJ, Chan M, Bhatti H, Halton M, Grassi L, Johansen C (2011). Prevalence of depression, anxiety, and adjustment disorder in oncological, haematological, and palliative-care settings: a meta-analysis of 94 interview-based studies. Lancet Oncol.

[10] Widera EW, Block SD (2012). Managing grief and depression at the end of life. Am Fam Physician.

[11] Magnil M, Gunnarsson R, Björkstedt K, Björkelund C (2008). Prevalence of depressive symptoms and associated factors in elderly primary care patients: A descriptive study. J Clin Psychiatry.

[12] O’Hara R, Coman E, Butters MA, Snyder PJ, Nussbaum PD, Robins DL (2006). Late-life depression. Clinical neuropsychology, a pocket handbook for assessment.

[13] King DA, Heisel MJ, Lyness JM (2005). Assessment and psychological treatment of depression in older adults with terminal or life threatening illness. Clinical Psychol (New York).

[14] Rayner L, Lee W, Price A, Monroe B, Sykes N, Hansford P (2011). The clinical epidemiology of depression in palliative care and the predictive value of somatic symptoms: cross-sectional survey with four-week follow-up. J Palliat Med.

[15] Bužgová R, Jarošová D, Hajnová E (2015). Assessing anxiety and depression with respect to the quality of life in cancer inpatients receiving palliative care. Eur J Oncol Nurs.

[16] Ohnsorge K, Gudat H, Rehmann-Sutter C (2014). What a wish to die can mean: reasons, meanings and functions of wishes to die, reported from 30 qualitative case studies of terminally ill cancer patients in palliative care. BMC Palliat Care.

[17] Smith TF, Steel K, Fries BE, Morris JN, Belleville-Taylor P, Curtin-Telegdi N, Frijters D, Szczerbińska K (2010). interRAI Palliative Care Palliative Care Assessment Form and User’s Manual. Version 9.1.

[18] Steel K, Ljunggren G, Topinková E, Morris JN, Vitale C, Parzuchowski J (2003). The RAI-PC: an assessment instrument for palliative care in all settings. Am J Hosp Palliat Care.

[19] Hirdes JP, Ljunggren G, Morris JN, Frijters DH, Soveri HF, Gray L (2008). Reliability of the interRAI suite of assessment instruments: a 12-country study of an integrated health information system. BMC Health Serv Res.

[20] Burrows AB, Morris JN, Simon SE, Hirdes JP, Phillips C (2000). Development of a minimum data set-based depression rating scale for use in nursing homes. Age Ageing.

[21] Fisher KA, Seow H, Brazil K, Freeman S, Smith TF, Guthrie DM (2014). Prevalence and risk factors of depressive symptoms in a Canadian palliative home care population: a cross-sectional study. BMC Palliat Care.

[22] Martin L, Poss JW, Hirdes JP, Jones RN, Stones MJ, Fries BE (2008). Predictors of a new depression diagnosis among older adults admitted to complex continuing care: implications for the depression rating scale (DRS). Age Aging.

[23] Koehler M, Rabinowitz T, Hirdes J, Stones M, Carpenter GI, Fries BE, et al. Measuring depression in nursing home residents with the MDS and GDS: An observational psychometric study. BMC Geriatr. 2005, doi: 10.1186/1471-2318-5-1. 10.1186/1471-2318-5-1 PMC546185 15627403

[24] Hirdes JP, Smith TF, Rabinowitz T, Yamauchi K, Pérez E, Telegdi NC (2002). The Resident Assessment Instrument-Mental Health (RAI-MH): Inter-rater reliability and convergent validity. J Behav Health Serv Res.

[25] Hirdes JP, Frijters DH, Teare GF (2003). The MDS CHESS Scale: A new measure to predict mortality in institutionalized older people. J Am Geriatr Soc.

[26] Morris JN, Fries BE, Mehr DR, Hawes C, Phillips C, Mor V (1994). MDS cognitive performance scale©. J Gerontol.

[27] Morris JN, Fries BE, Morris SA (1999). Scaling ADLs within the MDS. J Gerontol A Biol Sci Med Sci.

[28] Fries BE, Simon SE, Morris JN, Flodstrom C, Bookstein FL (2001). Pain in US nursing homes: validating a pain scale for the Minimum Data Set. Gerontologist.

[29] Hosmer DW, Lemeshow S, Sturdivant RX (2013). Applied logistic regression.

[30] Bewick V, Cheek L, Ball J (2005). Statistics review 14: Logistic regression. Crit Care.

[31] World Health Organization. ICD-10 Version: 2016. [http://apps.who.int/classifications/icd10/browse/2016/en#/II]

[32] Nissim R, Gagliese L, Rodin G (2009). The desire for hastened death in individuals with advanced cancer: a longitudinal qualitative study. Soc Sci Med.

[33] Chochinov HM, Wilson KG, Enns M, Mowchun N, Lander S, Levitt M (1995). Desire for death in the terminally ill. Am J Psychiatry.

[34] Rosenfeld B, Breitbart W, Galietta M, Kaim M, Funesti-Esch J, Pessin H (2000). The Schedule of Attitudes Toward Hastened Death: measuring desire for death in terminally ill cancer patients. Cancer.

[35] Breitbart W, Rosenfeld B, Pessin H, Kaim M, Funesti-Esch J, Galietta M (2000). Depression, hopelessness, and desire for hastened death in terminally ill patients with cancer. J Am Med Assoc.

[36] Kelly B, Burnett P, Pelusi D, Badger S, Varghese F, Robertson M (2003). Factors associated with the wish to hasten death: a study of patients with terminal illness. Psychol Med.

[37] Emanuel E, Fairclough D (2000). Desires related to euthanasia and physician assisted suicide among terminally ill patients. J Am Med Assoc.

[38] O’Mahony S, Goulet J, Kornblith A, Abbatiello G, Clarke B, Kless-Siegel S (2005). Desire for hastened death, cancer pain and depression: report of a longitudinal observational study. J Pain Symptom Manage.

[39] Mystakidou K, Rosenfeld B, Parpa E, Katsouda E, Tsilika E, Galanos A, Vlahos L (2005). Desire for death near the end of life: the role of depression, anxiety and pain. Gen Hosp Psychiatry.

[40] Covinsky KE, Goldman L, Cook EF, Oye R, Desbiens N, Reding D (1994). The impact of serious illness on patients’ families. J Am Med Assoc.

[41] Albert T, Williams GT, Legowski B, Remis R. The economic burden of HIV/AIDS in Canada. Canadian Policy Research Networks. 1998; [http://www.cprn.org/documents/18422_en.pdf.]

[42] Carpenito-Moyet LJ (2007). Understanding the nursing process: concept mapping and care planning for students.

[43] Neufeld E, Freeman S, Joling K, Hirdes JP (2014). “When the golden years are blue”: Changes in depressive symptoms over time among older adults newly admitted to long-term care facilities. Clin Gerontol.

[44] Ferrand E, Dreyfus JF, Chastrusse M, Ellien F, Lemaire F, Fischler M (2012). Evolution of requests to hasten death among patients managed by palliative care teams in France: A multicentre cross-sectional survey (DemandE). Eur J Cancer.

[45] Boswell EB, Stoudemire A (1996). Major depression in the primary care setting. Am J Med.

[46] Callahan CM (2001). Quality improvement research on late life depression in primary care. Med Care.

[47] Asghar-Ali A, Wagle K, Braun U (2013). Depression in terminally ill patients: dilemmas in diagnosis and treatment. J Pain Symptom Manage.

[48] Rosenfeld B, Pessin H, Marziliano A, Jacobson C, Sorger B, Abbey J (2014). Does desire for hastened death change in terminally ill cancer patients?. Soc Sci Med.

[49] Freeman S, Hirdes J, Stolee P, Garcia J, Smith TF, Steel K (2014). Care planning needs of palliative home care clients: Development of the interRAI palliative care assessment clinical assessment protocols (CAPs). BMC Palliat Care.

[50] Ontario Association of Community Care Access Centers: OACCAC News: Implementation of electronic palliative assessment tool supported by education. 2012, 11:5. [http://oaccac.com/News/Documents/OACCAC News Feb 2012.pdf]

